# Tricuspid Regurgitation in the Setting of Cardiac Implantable Electronic Devices

**DOI:** 10.1016/j.shj.2024.100319

**Published:** 2024-05-22

**Authors:** Omar M. Aldaas, Gary Ma, Quan Bui, Ryan R. Reeves, Ulrika Birgersdotter-Green

**Affiliations:** Division of Cardiology, University of California San Diego, La Jolla, California

**Keywords:** Cardiac implantable electronic devices, Cardiac resynchronization therapy, Defibrillator, Pacemaker, Tricuspid regurgitation

## Abstract

Cardiac implantable electronic devices (CIEDs) have been increasingly used in the management of various rhythm disorders amidst an aging population with more prevalent cardiovascular comorbidities. Although generally well-tolerated and safe, implantation of CIEDs may result in or worsen tricuspid regurgitation (TR), which is associated with a higher risk of morbidity and mortality. Several mechanisms of TR following device implantation have been proposed, and multiple diagnostic tests, percutaneous and surgical interventions, and alternative pacing methods have been developed to address this. CIED-related TR thus requires a multidisciplinary team of experts in cardiac imaging, interventional cardiology, electrophysiology, and cardiothoracic surgery with a comprehensive understanding of this multifaceted disease. The objective of this review is to summarize the epidemiology, clinical presentation, and management of CIED-related TR.

## Introduction

Cardiac implantable electronic devices (CIEDs) have been increasingly used in the management of various rhythm disorders amidst an aging population with more prevalent cardiovascular comorbidities. Over 730,000 permanent pacemakers (PPMs) and 330,000 implantable cardioverter defibrillators (ICDs), including cardiac resynchronization therapy (CRT) devices, are implanted annually worldwide.^1^ Although generally well-tolerated and safe, implantation of CIEDs may result in or worsen tricuspid regurgitation (TR), which is associated with a higher risk of heart failure and mortality.^2^^,^^3^ Several mechanisms of TR following CIED implantation have been postulated, which can broadly be categorized as implantation-, pacing-, and device-related. While the cause of CIED-induced TR is likely a multifaceted combination of all the above, there is no consensus on the predominant mechanism due to conflicting data. This makes it challenging to determine the optimal management strategy for these patients. However, novel interventional and surgical options have been developed to address these challenges.^4^ This multidisciplinary review article aims to review the epidemiology, clinical presentation and outcomes, diagnostic approaches, and management of CIED-related TR in light of the emerging transcatheter treatment solutions that have been recently approved by US Food and Drug Administration (FDA).

## Epidemiology and Clinical Outcomes

Significant TR (of at least moderate severity) is common and affects up to 12% of patients.^5^ Etiologies are typically categorized as primary (i.e., CIED-related, endocarditis, and myxomatous degeneration) or secondary (i.e., functional from atrial enlargement and dysfunction, left ventricular dysfunction, and pulmonary hypertension), with the latter accounting for the majority of cases (90%).^5^ After initial reports in the late 1900s, CIED-related TR has become increasingly recognized and involves complex management, leading to its proposal as a third distinct mechanism.^3^^,^^4^ The prevalence of CIED-related TR varies significantly with estimates between 7% and 40%.^4^^,^^6^^,^^7^ The variability is explained by retrospective studies with small sample sizes and short-term follow-up, different echocardiographic techniques, and inconsistent grading definitions of TR.^6^ One prospective study, including over 35,000 echocardiograms at ten Spanish medical centers, found that CIED-related TR represented 66% of the cases of primary TR and 5% of all cases of significant TR.^8^ Another prospective study found that the prevalence of moderate or severe CIED-related TR was 5% after CIED implantation.^9^ Further prospective studies with standard assessment methods and accepted TR grading criteria are needed to understand the true prevalence of this morbid condition.

The natural history of CIED-related TR follows that of other etiologies with progressive regurgitation, adverse right ventricular remodeling, pulmonary hypertension, heart failure, and increased mortality.^6^ Early ICD studies hinted at the increased risk of heart failure and death despite a reduction in sudden death.^4^^,^^7^ However, the possibility that an endocardial lead could cause significant TR and associated heart failure was not widely recognized at that time.^4^^,^^7^ Symptoms typically represent those of right heart failure, including dyspnea, hepatomegaly, ascites, and peripheral edema.^6^ With regard to outcomes, increasing TR severity, regardless of etiology, has been shown to be a significant predictor of mortality independent of biventricular function, pulmonary hypertension and atrial fibrillation.^5^^,^^10^ Similarly, CIED-related severe TR has been associated with a poor prognosis with increased risk of heart failure hospitalization, tricuspid valve intervention, CRT upgrade, and mortality.^11^^,^^12^ In a retrospective study of 18,800 patients with CIED, significant TR was greater in patients with CIED compared to those without (23.8 vs. 7.7%).^13^ Furthermore, this severity of TR was associated with a 1.6 to 2.5-fold increased risk of all-cause mortality.^13^ Also, in patients undergoing CIED, baseline right ventricular dilation was associated with worse TR and portends worse survival.^2^ These studies highlight that timely recognition and intervention of CIED-related TR may represent an opportunity to improve morbidity and mortality.

## Mechanisms of Tricuspid Regurgitation

**Figure 1:**
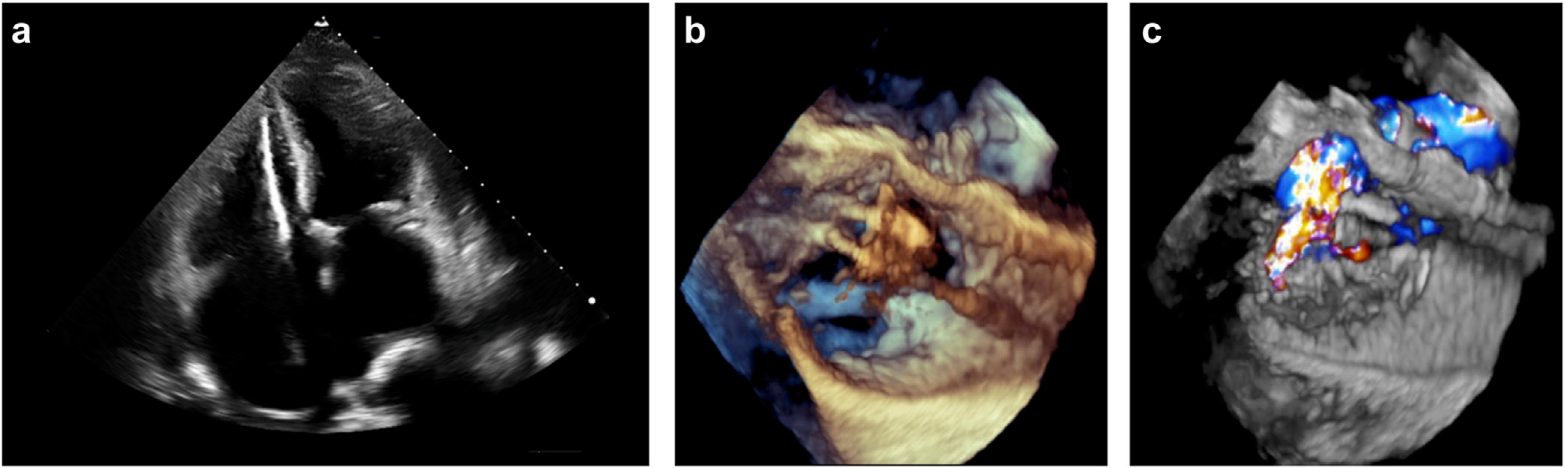
Echocardiography demonstrating device-related severe tricuspid regurgitation. (a) Apical 4 chamber view showing the ventricular lead adjacent to the septal leaflet of the tricuspid valve. (b) Three-dimensional echocardiographic view constructed from transesophageal images showing a surgeon’s view of the tricuspid valve with the device lead impinging on the septal leaflet with (c) resultant severe tricuspid regurgitation.

Transvenous PPMs, ICDs, and CRT devices are typically implanted with a lead that crosses the tricuspid valve and is fixated in the right ventricle. The posterior and septal leaflets of the tricuspid valve appear to be the most commonly affected by transvenous leads (Figure 1).^14^ Although demonstration of lead–leaflet interaction is sufficient to diagnose CIED-related TR, causality is rarely established. More often, CIED and TR coexist, and a causal relationship is assumed when new TR is diagnosed or pre-existing TR worsens following CIED implantation.

Mechanisms of CIED-related TR are categorized into implantation-related, pacing-related, and device-related (Table 1). Implantation technique is operator-dependent and has changed over time. Some argue that direct crossing of the tricuspid valve may result in less trauma to the tricuspid valve apparatus relative to the “prolapsing” technique, but this has not been extensively studied. Silicone-insulated leads, higher number of leads crossing the tricuspid annulus, presence of bulkier and stiffer defibrillator leads, and implant location in the apex instead of the right ventricular outflow tract have all been implicated as possible predictors of TR following CIED implantation.^15^, ^16^, ^17^, ^18^, ^19^, ^20^ However, the majority of these studies are small, retrospective studies, often with conflicting results.

**Table 1:** Potential mechanisms of cardiac implantable electronic device-related tricuspid regurgitation

Implantation	Pacing	Device
• “Prolapsing technique”∗	• Alteration of right ventricular geometry with right ventricular pacing, irrespective of pacing burden	• Adherence to leaflet • Perforation, laceration, or avulsion of leaflet • Interference with subvalvular apparatus
• Silicone insulate leads∗		
• Higher number of leads		
• Bulkier/stiffer defibrillator leads		
• Apical implant		

∗ Controversial due to a lack of, conflicting, or biased data.

While right ventricular pacing has been associated with worsening TR, a higher pacing burden has not been shown to correlate with this.^7^^,^^17^^,^^21^^,^^22^ It is thus thought to be an alteration of right ventricular geometry with pacing that can occur irrespective of pacing burden, which is in contrast to the worsening mitral regurgitation seen as a result of dyssynchronous contraction of the interventricular septum.^21^^,^^23^^,^^24^ Transvenous leads can also disrupt the tricuspid valve apparatus via impingement of or adherence to a leaflet, perforation, laceration, or avulsion of a leaflet, interference with the subvalvular apparatus, and predisposition to thrombus formation and endocarditis.^16^^,^^25^ Due to these unique mechanisms of TR, CIED-related TR has been reclassified from “primary” TR to its own separate etiologic entity to emphasize the distinct diagnostic approach and multidisciplinary management that it warrants.

## Diagnostic Approaches

Echocardiography remains a cornerstone in the evaluation of CIED-related TR. Echocardiography allows for detailed assessment of the tricuspid valve apparatus integrity, device lead interactions, and quantification of regurgitant severity by utilizing both 2D and, more recently, 3D imaging modalities. Furthermore, echocardiography plays a key role in assessing the impact of CIED-related TR on cardiac function and the associated hemodynamic consequences. Incorporation of advanced echocardiographic techniques, such as Doppler imaging and strain analysis, improves the sensitivity and specificity of diagnosing TR.^26^^,^^27^ The ability to dynamically visualize cardiac structures with ultrasound aids in identifying key mechanisms of TR discussed above.

It is for these reasons that candidates for CIED implantation should ideally have a comprehensive baseline transthoracic echocardiogram with a focus on the tricuspid valve and right ventricle prior to implantation, as this would inform an interdisciplinary discussion about whether valve-sparing strategies should be pursued instead. Moreover, it would facilitate timely recognition of new or worsening TR post-implant. Transesophageal echocardiography is the imaging modality of choice to assess CIED-related TR and help distinguish it from other incidental causes of TR. Incorporation of 3D echocardiography allows for more reliable visualization of the device lead, tricuspid valve, and mechanism of TR.^16^^,^^28^, ^29^, ^30^, ^31^, ^32^

Cardiac computed tomography can provide important complementary information, especially if transvenous lead extraction (TLE) is being considered. It can help assess lead fibrosis and vein stenosis and, in some instances, provide insight into the possible lead-leaflet interaction and mechanism of CIED-related TR that may not have been fully visualized by echocardiography.^33^^,^^34^ It is also useful in planning for implantable transcatheter valves and the need for lead jailing, as discussed below.^35^ However, a major limitation of cardiac computed tomography is the significant blooming artifact that can be caused by CIED leads, which makes interpretation challenging. Similarly, despite cardiac magnetic resonance imaging being shown to be safe for both conditional and nonconditional devices, it is significantly limited by artifacts related to CIED type and size (worst in CRT-defibrillators and subcutaneous ICDs). Visualization of the tricuspid leaflets is often difficult, but it may be possible to quantify right ventricular size, volumes, function, remodeling, fibrosis, and TR.^36^

## Medical Therapy

Diuretics remain central in the medical management of CIED-related TR and symptomatic right heart failure. Loop diuretics can be augmented with aldosterone antagonists, but data are lacking regarding the long-term outcomes of conservative medical management in CIED-related TR as more invasive strategies, discussed below, are often pursued.^37^ This is largely due to the challenge in increasing dosage of diuretics due to kidney dysfunction from cardiorenal syndrome or diuretic-induced acute kidney injury and hemodynamic instability from impaired cardiac output.

## Transvenous Lead Extraction

The decision to pursue TLE is complex and should involve a multidisciplinary team to try to predict the probability of improving or worsening TR and to weigh the risks of procedural complications. Despite the improving safety and efficacy of TLE, there are still no prospective data or guideline recommendations addressing TLE in severe TR in the absence of infection.^38^, ^39^, ^40^, ^41^ On one hand, CIED-related TR that is left untreated is associated with adverse right ventricular remodeling and tricuspid annular dilation, resulting in irreversible severe TR, as well as increased mortality estimated between 40 and 75%.^11^^,^^42^ On the other hand, some studies have reported that as little as 25% to 35% of patients had successful TLE and improvements in CIED-related TR.^43^^,^^44^ Furthermore, severe injuries to the tricuspid valve apparatus have been reported to occur in 2.5% of cases, but with any grade increase of TR occurring in as much as 11.5% of cases.^45^^,^^46^ This highlights the importance of a thorough assessment of the cause of TR in the presence of CIEDs. In patients where the TR is thought to be CIED-related and without significant right ventricular dysfunction or tricuspid annular dilation, TLE should be considered. Procedural volume and technical expertise are also important considerations. Consequently, it is crucial to refer these patients to specialized valve centers with a multidisciplinary heart team capable of meticulously weighing the risks and benefits. Although longer lead dwell time has been shown to be a risk factor for acute TR worsening after TLE, there are insufficient data regarding whether mechanical sheaths, laser sheaths, femoral snares, and other specific techniques are associated with the risk of worsening TR.^45^^,^^46^ The role of TLE to avoid lead jailing and facilitate percutaneous treatments discussed below remains unclear.

## Percutaneous Treatment

Percutaneous therapies to treat significant TR have recently gained significant attention, particularly given the expansion of available therapeutic options, including transcatheter edge-to-edge repair (TEER), transcatheter valve replacement, heterotopic bi-caval valve placement, and transcatheter annuloplasty. Many of these therapeutic options remain investigational with limited observational data or with small numbers of patients, and several challenges lie ahead regarding patient selection and procedural planning due to the complex structural milieu that results in significant TR. Furthermore, questions regarding the efficacy of these therapies for CIED-related TR remain unexplored. This is in part due to the fact that many randomized controlled trials exclude patients with CIED-related TR with lead impingement or adherence clearly demonstrated.

**Figure 2:**
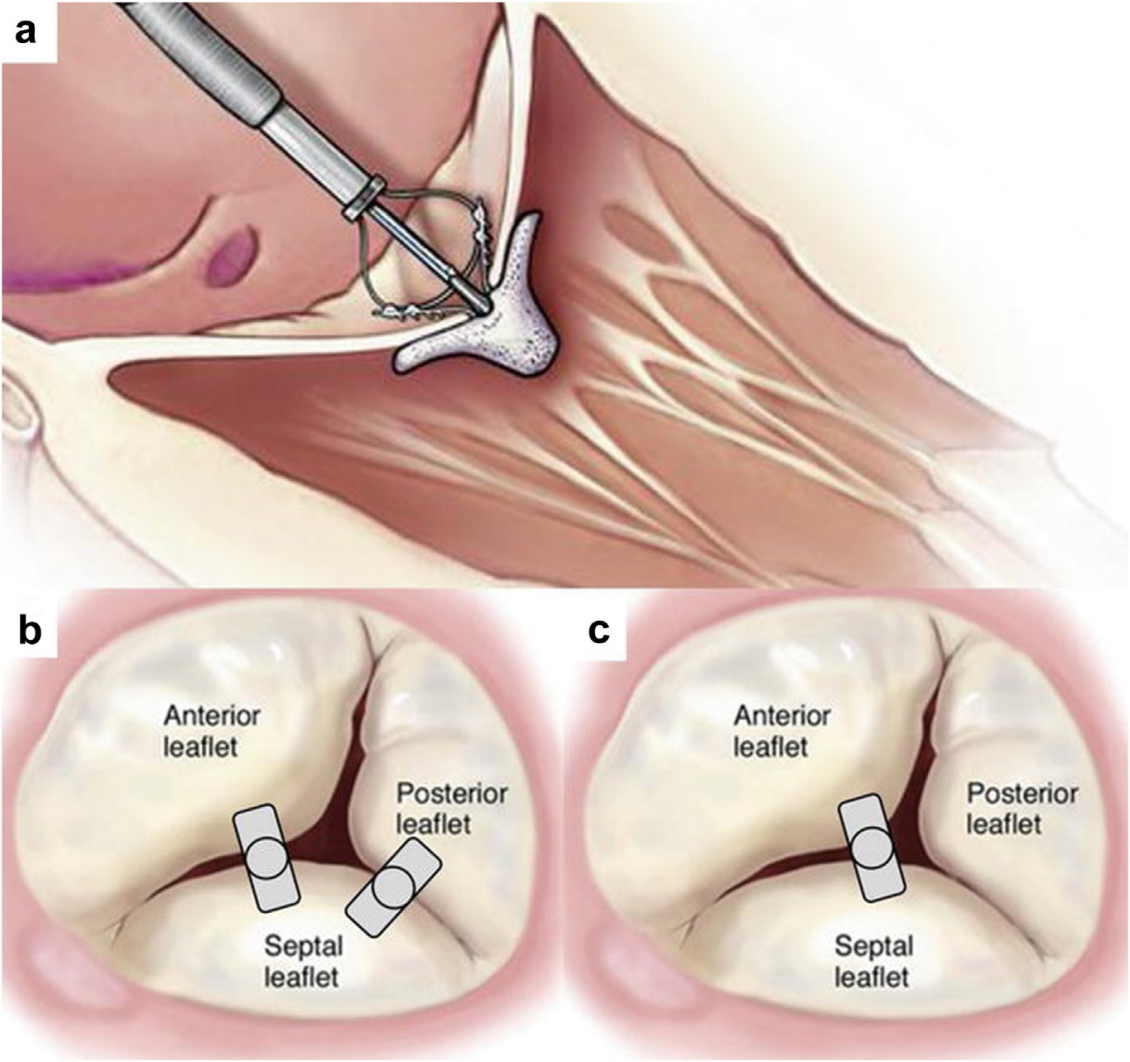
(a) Percutaneous tricuspid valve repair with MitraClip. (b) Triple-orifice technique. (c) Bicuspidization approach. Reproduced with permission.^65^

TEER is a well-established technique for mitral valve repair and has been utilized in the treatment of significant TR (Figure 2). TEER devices include the MitraClip and TriClip systems (Abbott Laboratories) and the PASCAL Precision System (Edwards Lifesciences). Use of the MitraClip system on the tricuspid valve has been reported in single-center and multinational registries since the approval for use on the mitral valve. Many of these nonrandomized studies report a reduction in the degree of TR as well as symptom, functional, and/or clinical improvement, while excluding patients with pacemaker lead-related TR. As an example, a propensity-matched case-control study from the TriValve registry (22 European and North American centers) evaluated multiple different transcatheter tricuspid valve therapies, with the MitraClip being the dominant system studied.^47^ Overall survival and survival free from heart failure were significantly higher in the TEER group. A right ventricular lead was present five times more often in the TEER group than in the control group, while the benefit of TEER was not as robust in the presence of a a right ventricular lead.

**Figure 3:**
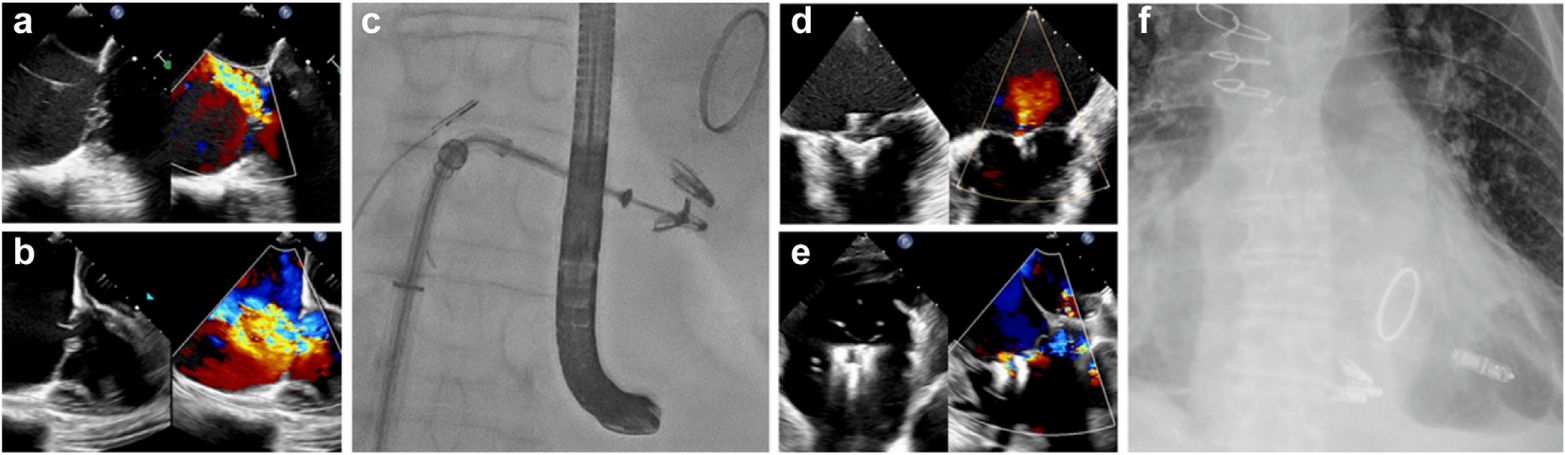
A case representative of the challenges presented with CIED and tricuspid regurgitation. A 91-year-old male with atrial fibrillation, a mechanical mitral valve, and a pacemaker with a single right ventricular lead developed severe tricuspid regurgitation and congestive hepatopathy-related cirrhosis. He was referred for lead extraction, placement of a leadless pacemaker, and ultimately TEER of the tricuspid valve with three MitraClip devices. (a) Pre-extraction TEE revealing severe TR. (b) Postextraction TEE revealing disrupted tricuspid leaflets and severe TR. (c) Fluoroscopy during the TEER procedure showing placement of the second clip under TEE and ICE guidance. (d) ICE imaging showing two tricuspid leaflets being captured by a MitraClip and ultimately trace TR. (e) Final TEE imaging showing the three MitraClips, pulling all three leaflets together, and the resulting trace TR. (f) Final chest X-ray showing the three MitraClips and the leadless pacemaker Abbreviations: CIED, cardiac implantable electronic device; ICE, intracardiac echocardiography; TEE, trans-esophageal echocardiography; TEER, transcatheter edge-to-edge repair; TR, tricuspid regurgitation.

The TriClip device recently gained approval for use in the United States, and the TriClip and PASCAL systems have received CE MARK. The TRILUMINATE study reported on 350 patients randomized 1:1 to TriClip and medical therapy vs. medical therapy alone.^48^^,^^49^ Safety was established with 98.3% freedom from major adverse events at 30 days, the degree of TR was significantly reduced and sustained to 1 ​year, and health status was markedly improved as determined by survival and Kansas City Cardiomyopathy Questionnaire scores. Patients were excluded if device leads were present that would prevent appropriate placement of the TriClip device, and 16% of patients in the TEER group had a CIED in place. At 1 ​year, 3% of patients in each group required placement of a CIED, suggesting that tricuspid TEER does not carry a risk of conduction disturbance. A common theme in the literature is a subjective and ill-defined assessment of the role a trans-tricuspid device lead may play during a transcatheter intervention for TR and a lack of statistics regarding the exclusion of patients due to the presence of CIEDs. It should be noted that the presence of any lead through the tricuspid valve can pose a challenge to tricuspid TEER due to both the presence of the lead in the jet area and the shadowing artifact that can make imaging guidance suboptimal (especially for device placement posterior to the lead). A representative case of CIED-related severe TR resulting in lead extraction and tricuspid TEER is presented in Figure 3. Additional studies are required to examine how to proceed with tricuspid TEER in the presence of CIED, regardless of whether the TR is induced by the CIED, whether TEER can be utilized in combination with TLE, and whether there are long-term interactions when both CIED leads and TEER devices are present.

Transcatheter tricuspid valve replacement (TTVR) in patients with recurrent tricuspid valve disease (regurgitation or stenosis) after surgical bioprosthetic valve replacement or annuloplasty-based repair has been performed since the availability of balloon-expandable transcatheter valves. The vast majority of bioprosthetic valves will accommodate the balloon expandable S3 valve (Edwards Lifesciences), whereas valve-in-ring procedures are limited to specific characteristics of the annuloplasty device. Flexible or semirigid, complete rings are preferred over rigid, incomplete rings and bands. Use of the S3 valve system is approved for valve-in-valve/ring procedures in the mitral position but remains off-label in the tricuspid position. These procedures may be performed with a a right ventricular lead in place; however, differences in outcomes related to jailed leads have not been defined. In the case of valve-in-ring TTVR, the presence of a right ventricular lead may increase the stability of the deployed valve when the ring is flexible or incomplete.

**Figure 4:**
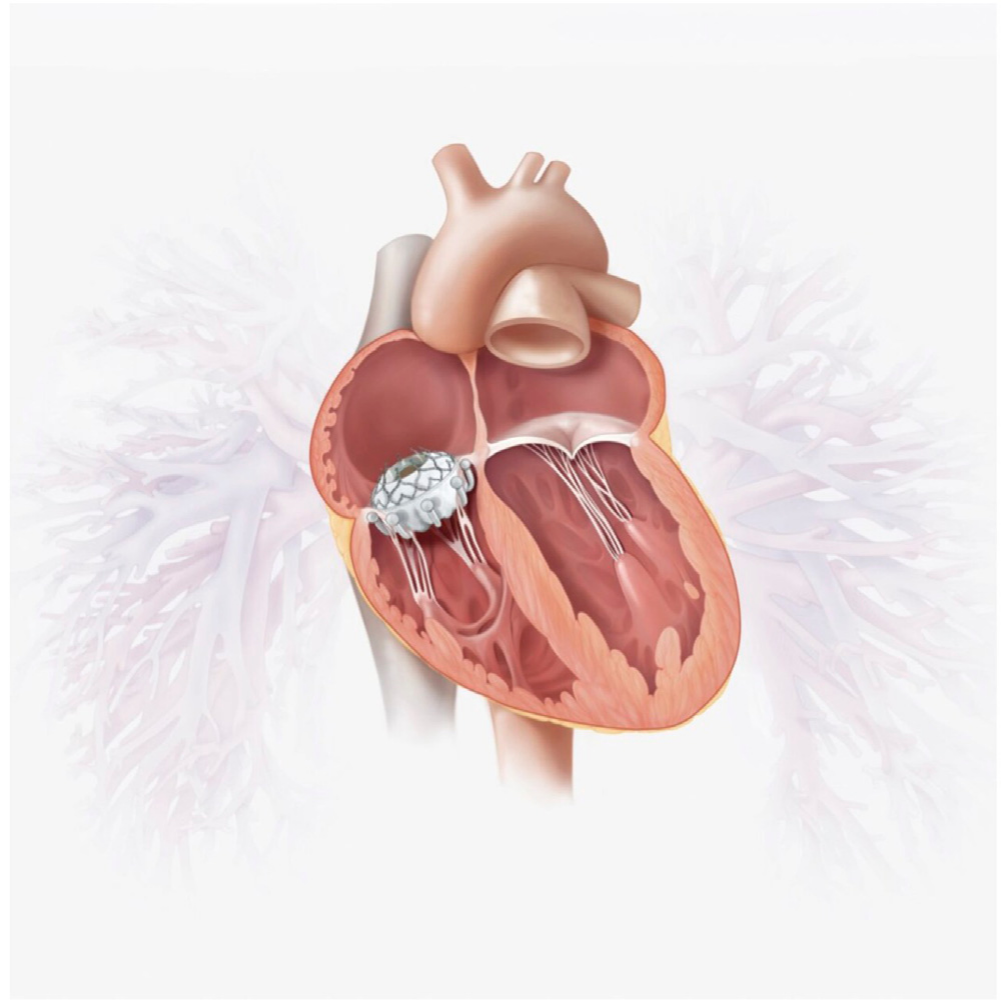
Schematic illustration of the EVOQUE transcatheter tricuspid valve. Image was reproduced with permission from Edwards Lifesciences Corporation.

TTVR in the native tricuspid annulus is a novel therapy that combines elements of annuloplasty and leaflet approximation to provide treatment for TR. The EVOQUE tricuspid replacement valve system (Edwards Lifesciences) is being studied in the TRISCEND II pivot trial (Figure 4).^50^^,^^51^ Data on the first 150 patients was presented at the Transcatheter Cardiovascular Therapeutics 2023 conference (San Francisco), including significant symptom, functional status, and TR improvement in the device arm with an acceptable safety profile. This led to US FDA approval for patients on optimal medical therapy with severe TR and right-sided heart failure. Exclusion criteria included dependency on a trans-tricuspid lead without an alternative pacing option, recent pacemaker placement, and the prior delivery of appropriate ICD therapy. Statistics regarding CIED-related exclusion have yet to be reported. The protocol mandated a plan in place prior to the procedure for placement of either a coronary sinus lead or a leadless pacemaker in the 36.5% of patients enrolled in the device arm with a pre-existing pacemaker or ICD. Furthermore, 14.7% of patients required the placement of a pacemaker after receiving the EVOQUE valve. The trial estimates an enrollment of 1070 subjects with a 5-year follow-up.

**Figure 5:**
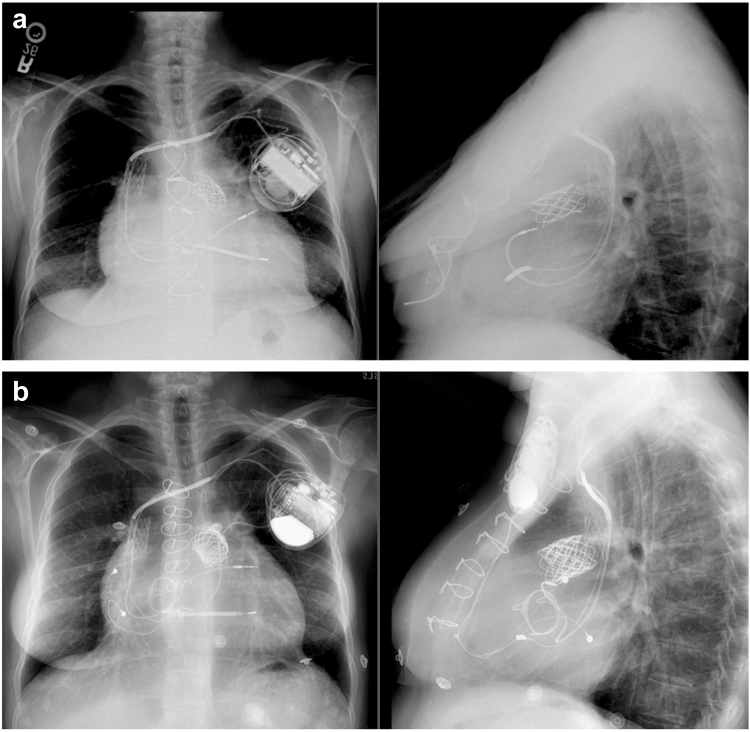
(a) Posteroanterior/lateral chest X-ray from a patient with tetrology of Fallot with atrial and ventricular arrhythmias who had a hybrid endocardial and epicardial defibrillator implanted. There are also superior vena cava, left pulmonary artery, and right ventricular outflow tract stents with a transcaether pulmonary valve replacement present. (b) Posteroanterior/lateral chest X-ray after a bioprosthetic tricuspid valve replacement (27 mm Magna Ease) demonstrating a defibrillator lead jailed by both the tricuspid valve replacement and the superior vena cava stent.

With both native and valve-in-valve/ring TTVR, the presence of an unextracted lead will result in the inevitable “jailing” of right ventricular leads adjacent to the implanted valve. While patients in the first human study for EVOQUE with jailed leads were not found to have significant impacts on lead function, follow-up included data only up to 1 year.^50^ The long-term implications of jailed leads will need to be studied, particularly given the challenges posed by transcatheter tricuspid valve replacement on TLE and/or placement of new leads across the replaced valve (Figure 5). This is particularly problematic in the setting of device-related infections, where jailed leads can significantly complicate or preclude TLE.

Direct transcatheter annuloplasty is designed to address tricuspid annular dilation, a common mechanism for significant TR, but may also be involved in the pathogenesis of CIED-induced TR. A variety of devices and techniques are currently in development to enable annular reduction through transcatheter means, with the aim of restoring tricuspid valve geometry and thereby improving valve function. The Cardioband (Edwards Lifesciences), TriAlign device (Mitralign Inc), and TriCinch (4Tech Cardio) are all investigational transcatheter devices, though only the Cardioband has been approved for use in European markets. Published data with the use of these therapies is limited to early feasibility in patients with functional TR, and therefore the use of these devices in the setting of CIED-induced TR is unstudied.^52^, ^53^, ^54^ However, as these technologies and techniques mature, transcathether annuloplasty may offer an additional tool in the armamentarium for treating CIED-related TR that may avoid the potential issues of jailed leads.

Finally, heterotopic caval valve implantation is an emerging treatment approach for patients with severe TR that involves implanting separate valve(s) upstream in either or both vena cavae, preserving the native tricuspid valve architecture. This strategy is not specifically designed to reduce TR, with focus rather on treating congestion associated with long-term severe TR and right ventricular failure. Preliminary studies on the various devices being developed with this approach indicate encouraging improvements in both quality of life and functional classification.^53^ Advantages of caval valve implantation involve avoiding interventions on complex native valve anatomy and serving as a rescue procedure in the event of failed TEER or annuloplasty in patients with prohibitive surgical risk. However, virtually all implantable caval valve devices will necessitate jailing of both the right atrial and ventricular leads, making this approach perhaps less favorable unless modular leadless pacemakers are considered simultaneously.

## Surgical Treatment

Tricuspid valve surgery is typically considered in patients who remain symptomatic despite diuretic therapy or in patients with evidence of progressive right ventricular dilation or dysfunction. However, the strength of these recommendations is discrepant between the American and European guidelines, highlighting the clinical ambiguity surrounding this.^55^^,^^56^ Data regarding the surgical management of CIED-related TR is lacking and sometimes conflicting. In a retrospective study of 116 patients with a PPM lead undergoing tricuspid valve annuloplasty, there was a 5-year freedom of 93.4% from tricuspid valve-related reoperation, 30-day mortality was 14.6%, and 5-year survival was 45%.^57^ The same group retrospectively reported a 30-day mortality of 6.3% and 5-year survival of 58% in a more contemporary cohort of 80 patients with at least one permanent right ventricular lead undergoing tricuspid valve repair or replacement.^58^ However, in a larger retrospective study with 622 patients that underwent tricuspid valve surgery in the presence of PPMs, 30-day mortality was 4.4% in CIED-related TR (n = 349) vs. 9.5% in CIED-associated TR (n = 249), which was a statistically significant difference.^59^

**Figure 6:**
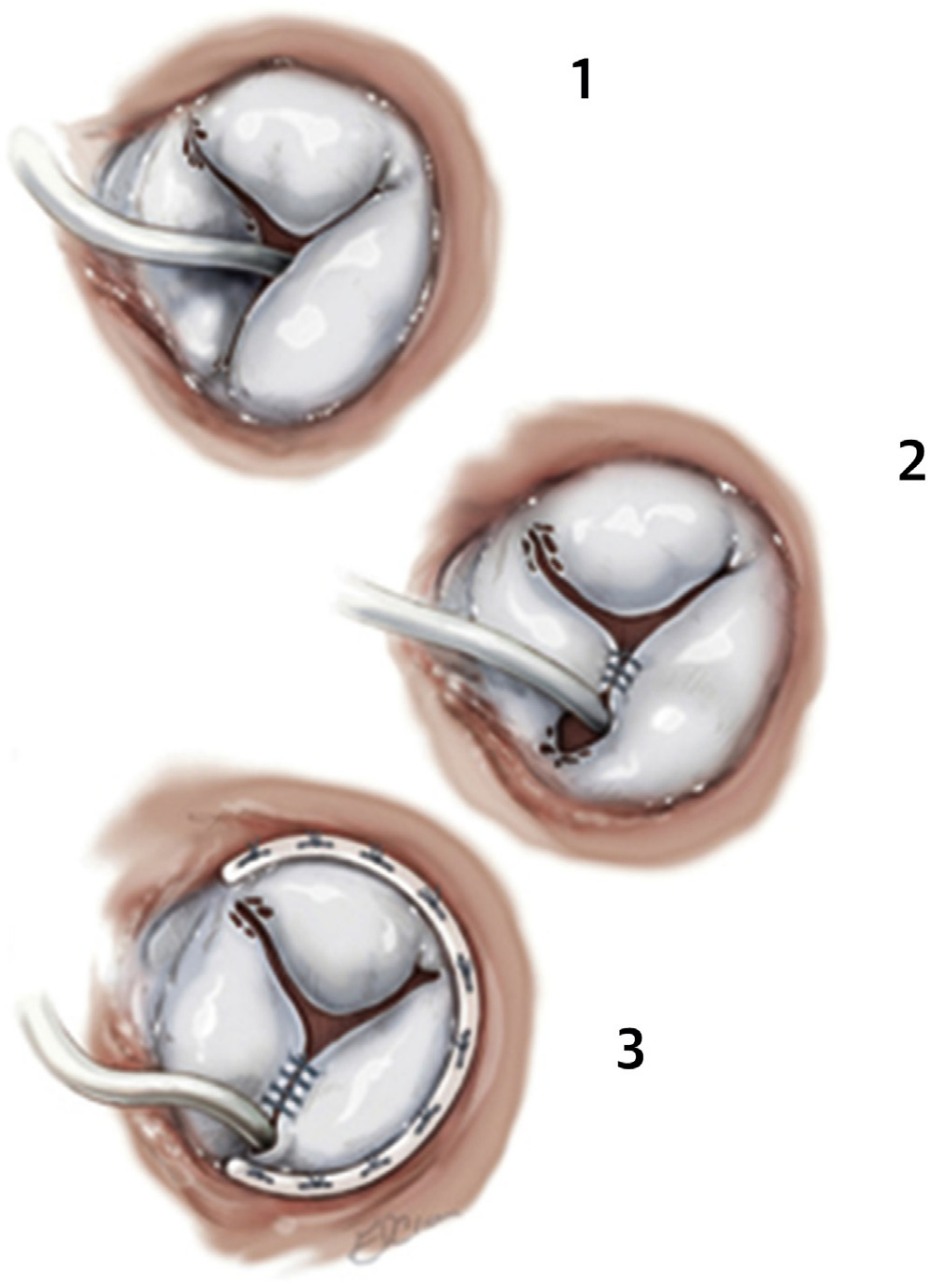
Steps of tricuspid valve repair. (1) Mobilized lead and leaflets of the tricuspid valve. (2) Lead repositioned in the cleft between the septal and inferior and posterior leaflets, with suture approximation of the leaflets above the cleft. (3) Repositioned lead with cleft closure and tricuspid valve annuloplasty.^66^

The high rate of early mortality that has historically been associated with tricuspid valve surgery has been mitigated to some extent with preoperative patient optimization and selection, especially in those operated on earlier in the disease course.^60^^,^^61^ It is thus important to risk stratify to select appropriate patients, optimize the right ventricle, and address CIED-related TR early in the disease. If surgery is pursued in CIED-related TR, both the device lead and tricuspid valve apparatus need to be addressed, as leads may adhere to, restrict, or perforate valve leaflets or dislocate during the operation.^16^ In some cases, the lead-related valve damage can be repaired with a suture or a patch, and an annuloplasty can be performed (Figure 6). However, the valve may need to be replaced if valve damage is extensive.^59^ The lead can either be repositioned in a location that does not interfere with valve function (usually the posteroseptal or the anteroposterior commissure) or extracted and replaced by a pacing method that does not cross the tricuspid valve (e.g., leadless pacing, coronary sinus, or epicardial).^59^^,^^62^^,^^63^

## Alternative Pacing Methods

**Figure 7:**
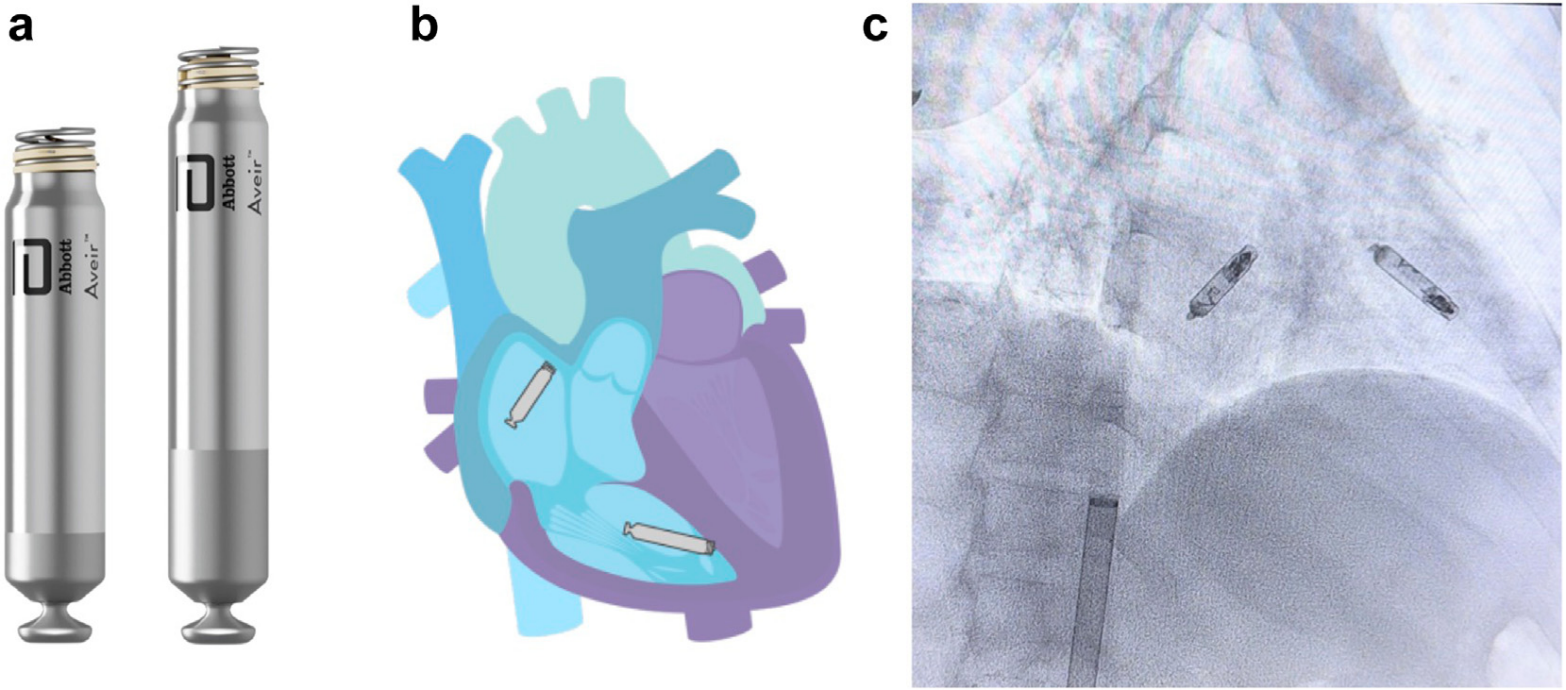
Aveir dual-chamber leadless pacemaker. (a) Atrial (left) and ventricular (right) Aveir leadless pacemaker devices are shown. (b) Schematic representation of the Aveir dual-chamber leadless pacemaker system once implanted. (c) An anterior-posterior chest X-ray projection showing the Aveir dual-chamber leadless pacemaker system immediately postimplant. Images reproduced with permission from Abbott Laboratories.

**Figure 8:**
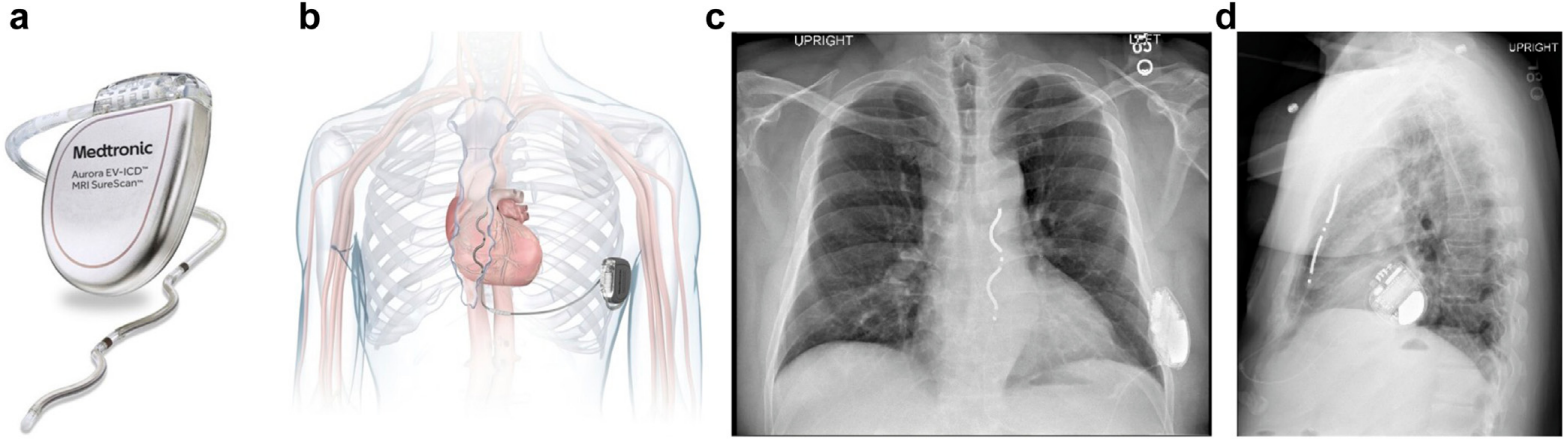
Aurora extra-vascular implantable cardioverter defibrillator is shown (a) *ex vivo* and (b) schematically following implantation. (c) and (d) show posterior-anterior and lateral projections, respectively, in a patient with the Aurora device implanted. Images used with permission from Medtronic, plc, 2024.

**Figure 9:**
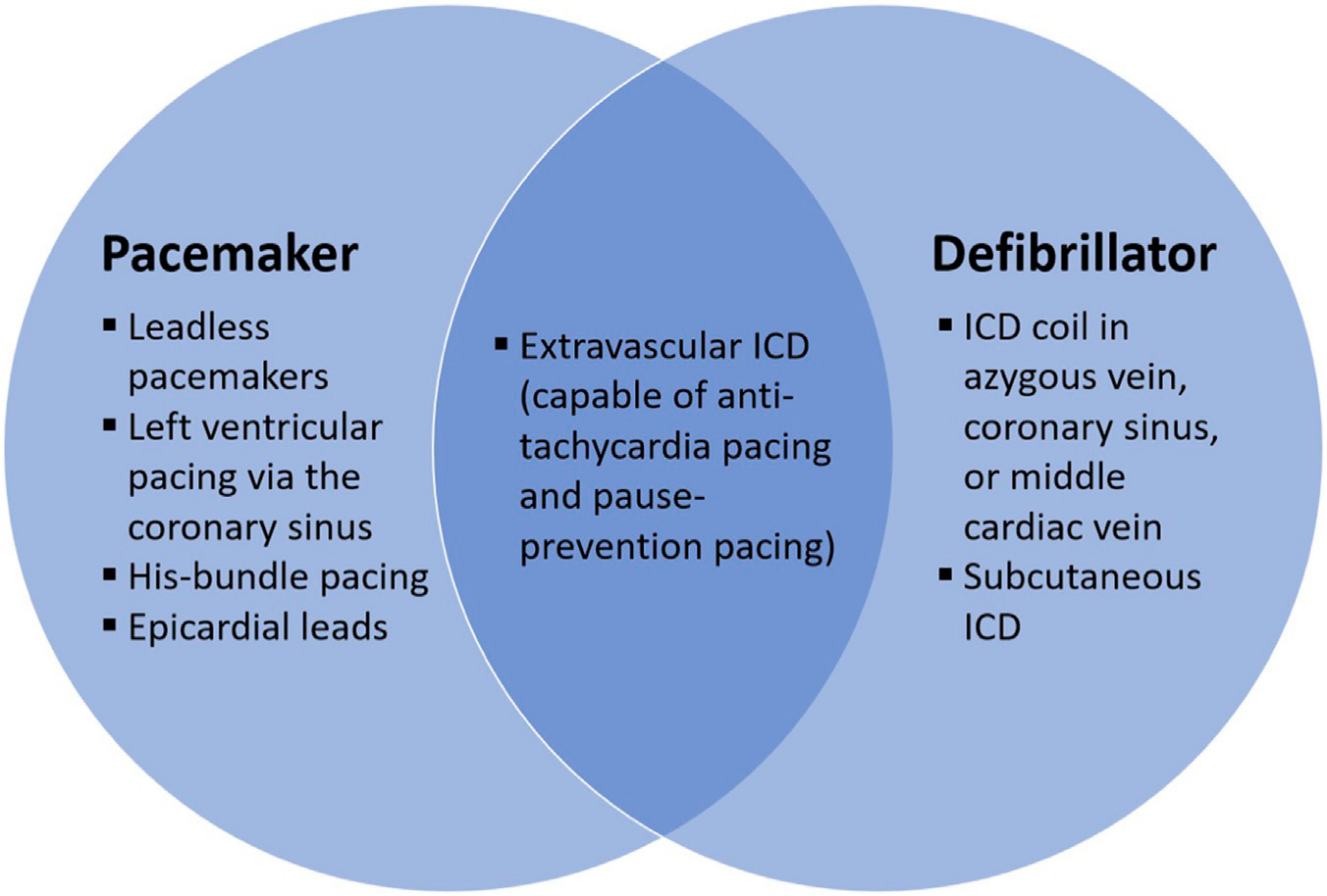
Tricuspid valve sparring options for cardiac implantable electronic devices that can pace and/or defibrillate Abbreviation: ICD, implantable cardioverter defibrillator.

Alternative pacing strategies that preserve valve function should be considered if ventricular pacing or defibrillation is deemed necessary following lead removal for CIED-related TR. If pacing is required, tricuspid valve-sparing options include leadless cardiac pacemakers (Figure 7), left ventricular pacing via the coronary sinus, His-bundle pacing from the atrial aspect of the tricuspid annulus, or surgical placement of epicardial leads. If a tricuspid-valve sparing device capable of defibrillation is required, standalone ICD coil in the azygous vein, coronary sinus, or middle cardiac vein, subcutaneous ICDs, and extra-vascular ICDs (Figure 8) can be considered. The extra-vascular ICD can also deliver antitachycardia and pause-prevention pacing (Figure 9). Deciding between these various options is complex and needs to take into account left ventricular ejection fraction, sinus rhythm rates, arrhythmia burden, presence of a wide QRS secondary to left bundle branch block, venous patency, prior open-heart surgery, frailty, body mass index, and many other factors.

## Summary

Improvements in diagnostic tests have resulted in CIED-related TR becoming increasingly recognized as a progressive disease with significant morbidity and mortality. Many mechanisms for CIED-related TR have been proposed. The complex pathophysiology of this condition is mirrored in the myriad of medical, percutaneous, and surgical options that have been employed to try to address this. Novel and diverse transcatheter treatment solutions have the potential to rapidly change the landscape of CIED management in the setting of TR. Additionally, alternative pacing methods have been developed that may reduce or avoid this complication all together. It thus necessitates a multidisciplinary team comprising experts in cardiac imaging, interventional cardiology, electrophysiology, and cardiothoracic surgery with a comprehensive understanding of these complexities.^64^

## Funding

The authors have no funding to report.

## Disclosure Statement

The authors report no conflict of interest.
